# Genome sequence of the Antarctic rhodopsins-containing flavobacterium *Gillisia limnaea* type strain (R-8282^T^)

**DOI:** 10.4056/sigs.3216895

**Published:** 2012-10-10

**Authors:** Thomas Riedel, Brittany Held, Matt Nolan, Susan Lucas, Alla Lapidus, Hope Tice, Tijana Glavina Del Rio, Jan-Fang Cheng, Cliff Han, Roxanne Tapia, Lynne A. Goodwin, Sam Pitluck, Konstantinos Liolios, Konstantinos Mavromatis, Ioanna Pagani, Natalia Ivanova, Natalia Mikhailova, Amrita Pati, Amy Chen, Krishna Palaniappan, Miriam Land, Manfred Rohde, Brian J. Tindall, John C. Detter, Markus Göker, James Bristow, Jonathan A. Eisen, Victor Markowitz, Philip Hugenholtz, Nikos C. Kyrpides, Hans-Peter Klenk, Tanja Woyke

**Affiliations:** 1 HZI – Helmholtz Centre for Infection Research, Braunschweig, Germany; 2 DOE Joint Genome Institute, Walnut Creek, California, USA; 3 Los Alamos National Laboratory, Bioscience Division, Los Alamos, New Mexico, USA; 4 Biological Data Management and Technology Center, Lawrence Berkeley National Laboratory, Berkeley, California, USA; 5 Oak Ridge National Laboratory, Oak Ridge, Tennessee, USA; 6 Leibniz Institute DSMZ - German Collection of Microorganisms and Cell Cultures, Braunschweig, Germany; 7 University of California Davis Genome Center, Davis, California, USA; 8 Australian Centre for Ecogenomics, School of Chemistry and Molecular Biosciences, The University of Queensland, Brisbane, Australia

**Keywords:** aerobic, motile, rod-shaped, moderately halotolerant, psychrophilic, chemoheterotrophic, proteorhodopsin, microbial mat, yellow-pigmented, *Flavobacteriaceae*, GEBA

## Abstract

*Gillisia limnaea* Van Trappen et al. 2004 is the type species of the genus *Gillisia*, which is a member of the well characterized family *Flavobacteriaceae*. The genome of *G. limnea* R-8282^T^ is the first sequenced genome (permanent draft) from a type strain of the genus *Gillisia*. Here we describe the features of this organism, together with the permanent-draft genome sequence and annotation. The 3,966,857 bp long chromosome (two scaffolds) with its 3,569 protein-coding and 51 RNA genes is a part of the *** G****enomic* ***E****ncyclopedia* of ***Bacteria***** and ***Archaea***** project.

## Introduction

Strain R-8282^T^ (= DSM 15749 = LMG 21470 = CIP 108418) is the type strain of the species *Gillisia limnaea* [1], which in turn is the type species of the *Gillisia*, a genus currently encompassing six known species [1]. The strain was isolated from a microbial mat in Lake Fryxell, Antarctica [1] during the MICROMAT project, which systematically collected novel strains from Antarctic lakes [2]. The genus was named after the Belgian bacteriologist Monique Gillis for her work on bacterial taxonomy [1]. The species epithet was derived from the Neo-Latin adjective ‘limnaeae’, living in the water, referring to the microbial mats in Lake Fryxell where the organism was first isolated [1]. PubMed records do not indicate any follow-up research with strain R-8282^T^ after the initial description and valid publication of the new species name *G. limnaea*, and genus *Gillisia* [1]. Here we present a summary classification and a set of features for *G. limnaea* R-8282^T^, together with the description of the genomic sequencing and annotation.

## Classification and features

A representative genomic 16S rRNA sequence of *G. limnaea* R-8282^T^ was compared using NCBI BLAST [3,4] under default settings (e.g., considering only the high-scoring segment pairs (HSPs) from the best 250 hits) with the most recent release of the Greengenes database [5] and the relative frequencies of taxa and keywords (reduced to their stem [6]) were determined, weighted by BLAST scores. The most frequently occurring genera were *Flavobacterium* (80.2%), *Gillisia* (17.8%), *Chryseobacterium* (1.0%) and *Cytophaga* (1.0%) (94 hits in total). Regarding the single hit to sequences from members of the species, the average identity within HSPs was 99.1%, whereas the average coverage by HSPs was 98.2%. Regarding the five hits to sequences from other members of the genus, the average identity within HSPs was 95.6%, whereas the average coverage by HSPs was 94.3%. Among all other species, the one yielding the highest score was *Gillisia hiemivivida* (AY694006), which corresponded to an identity of 97.1% and an HSP coverage of 90.8%. (Note that the Greengenes database uses the INSDC (= EMBL/NCBI/DDBJ) annotation, which is not an authoritative source for nomenclature or classification.) The highest-scoring environmental sequence was EU735617 (Greengenes short name: 'archaeal structures and pristine soils China oil contaminated soil Jidong Oilfield clone SC78'), which showed an identity of 99.0% and an HSP coverage of 98.4%. The most frequently occurring keywords within the labels of all environmental samples which yielded hits were 'librari' (3.2%), 'dure' (3.0%), 'bioremedi, broader, chromat, groundwat, microarrai, polylact, sampl, stimul, subsurfac, typic, univers' (2.9%), 'spring' (2.5%) and 'soil' (2.4%) (156 hits in total). The most frequently occurring keywords within the labels of those environmental samples which yielded hits of a higher score than the highest scoring species were 'soil' (15.4%), 'archaeal, china, contamin, jidong, oil, oilfield, pristin, structur' (7.7%) and 'antarct, cover, lake' (7.7%) (2 hits in total). Whereas some of these keywords confirm the environment of *G. limnaea*, others are indicative of other habitats in which related taxa are found.

**Figure 1:**
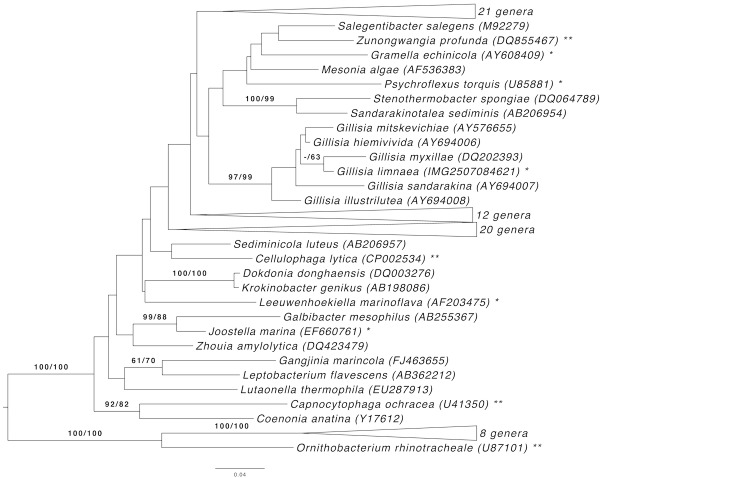
Phylogenetic tree highlighting the position of *G. limnaea* relative to the type strains of the type species of the genera within the family *Flavobacteriaceae*. The tree was inferred from 1,366 aligned characters [7,8] of the 16S rRNA gene sequence under the maximum likelihood (ML) criterion [9]. Rooting was done initially using the midpoint method [10] and then checked for its agreement with the current classification (Table 1). The branches are scaled in terms of the expected number of substitutions per site. Numbers adjacent to the branches are support values from 1,000 ML bootstrap replicates [11] (left) and from 1,000 maximum-parsimony bootstrap replicates [12] (right) if larger than 60%. Lineages with type strain genome sequencing projects registered in GOLD [13] are labeled with one asterisk, those also listed as 'Complete and Published' with two asterisks [14-16]; for *Ornithobacterium rhinotracheale* see CP003283)

Figure 1 shows the phylogenetic neighborhood of *G. limnaea* in a 16S rRNA based tree. The sequences of the two 16S rRNA gene copies in the genome differ from each other by up to eleven nucleotides, and differ by up to eight nucleotides from the previously published 16S rRNA sequence (AJ440991), which contains seven ambiguous base calls.

**Table 1:** Classification and general features of *G. limnaea* G-8282^T^ according to the MIGS recommendations [17] and NamesforLife [18].

**MIGS ID**	**Property**	**Term**	**Evidence code**
		Domain *Bacteria*	TAS [19]
		Phylum *Bacteroidetes*	TAs [20,21]
		Class *Flavobacteria*	TAS [22-24]
	Current classification	Order *Flavobacteriales*	TAS [21,25]
		Family *Flavobacteriaceae*	TAS [26-29]
		Genus *Gillisia*	TAS [1]
		Species *Gillisia limnaea*	TAS [1]
		Type strain R-8282	TAS [1]
	Gram stain	negative	TAS [1]
	Cell shape	rod-shaped	TAS [1]
	Motility	gliding motility likely, but not proven	NAS
	Sporulation	none	TAS [1]
	Temperature range	psychrophile, 5-30°C	TAS [1]
	Optimum temperature	20°C	TAS [1]
	Salinity	0-5% NaCl (w/v)	TAS [1]
MIGS-22	Oxygen requirement	aerobe	TAS [1]
	Carbon source	yeast extract, peptone	TAS [1]
	Energy metabolism	chemoheterotrophic, phototrophic	TAS [1]
MIGS-6	Habitat	fresh water	TAS [1]
MIGS-15	Biotic relationship	free living	TAS [1]
MIGS-14	Pathogenicity	none	NAS
	Biosafety level	1	TAS [30]
MIGS-23.1	Isolation	microbial mats	TAS [1]
MIGS-4	Geographic location	Lake Fryxell, McMurdo Dry Valleys, Antarctica	TAS [1]
MIGS-5	Sample collection time	between November 1998 and February 2001	TAS [1,2]
MIGS-4.1	Latitude	-77.614	NAS
MIGS-4.2	Longitude	163.184	NAS
MIGS-4.3	Depth	not reported	
MIGS-4.4	Altitude	not reported	

Evidence codes - TAS: Traceable Author Statement (i.e., a direct report exists in the literature); NAS: Non-traceable Author Statement (i.e., not directly observed for the living, isolated sample, but based on a generally accepted property for the species, or anecdotal evidence). Evidence codes are from the Gene Ontology project [31].

**Figure 2:**
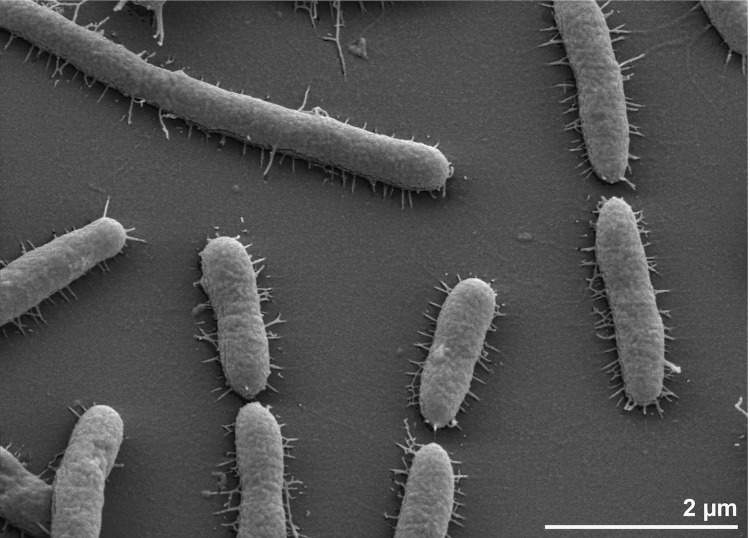
Scanning electron micrograph of *G. limnaea* R-8282^T^

Cells of strain *G. limnaea* R-8282^T^ are Gram-negative and rod-shaped [Figure 2] [1]. They are 0.7 µm in width and 3.0 µm in length [1], whereas scanning electron micrographs of strain R-8282^T^ revealed a cell diameter that varies from 0.4 µm to 0.5 µm, and a length that varies from 1.6 µm to longer than 4.9 µm [Figure 2], which is more consistent with data previously reported for several *Gillisia* strains [32-34]. Motility, especially gliding motility, was not observed [1], despite the presence of numerous genes associated with gliding motility (see below), and the presence of pili-containing cells in scanning electron micrographs of strain R-8282^T^. It is unclear if these pili are involved in gliding motility or bacterial adhesion to surfaces. Cells are strictly aerobic, psychrophilic and chemoheterotrophic [1]. Growth occurs between 5°C and 30°C with an optimum at 20°C [1]; the strain is unable to grow at temperatures above 37°C [1]. Growth occurs within a salinity range of 0% to 5% NaCl, but not in 10% NaCl, indicating moderate halotolerance [1]. Peptone and yeast extract were required for growth [1]. When cultivated on marine agar, colonies are yellow in color, convex and translucent with diameters of 1-3 mm forming entire margins after 6 days of incubation [1]. When cultivated on Anacker & Ordal’s agar, colonies become flat and round with entire margins and 0.7 to 0.9 mm in diameter after 14 days incubation [1]. Additionally growth is both detectable on nutrient agar and R2A, but the strain does not grow on trypticase soy agar [1]. Further detailed physiological data such as carbon source utilization, carbon degradation, and enzyme activities have been reported previously [1].

### Chemotaxonomy

The principal cellular fatty acids of strain R-8282^T^ are *iso*-C_15:0_, *anteiso*-C_15:0_, *iso*-C_15:1_, *iso*-C_16:0_, C_17:0 2-OH_, *iso*-C_17:0 3-OH_, *iso*-C_17:1 ω9c_, *anteiso*-C_17:1 ω9c_ and summed feature 3 (containing *iso*-C_15:0 2-OH_ and/or C_16:1 ω7c_) [1]. The major polar lipids were not reported for strain R-8282^T^.

## Genome sequencing and annotation

### Genome project history

This organism was selected for sequencing on the basis of its phylogenetic position [35], and is part of the *** G****enomic* ***E****ncyclopedia of* ***Bacteria**** and* ***Archaea***** project [36]. The genome project is deposited in the Genomes On Line Database [13] and the complete genome sequence is deposited in GenBank. Sequencing, finishing and annotation were performed by the DOE Joint Genome Institute (JGI). A summary of the project information is shown in Table 2.

**Table 2:** Genome sequencing project information

**MIGS ID**	**Property**	**Term**
MIGS-31	Finishing quality	Non-contiguous
MIGS-28	Libraries used	Four genomic libraries: one 454 pyrosequence standard library, two 454 PE libraries (4 kb and 10 kb insert size), one Illumina library
MIGS-29	Sequencing platforms	Illumina GAii, 454 GS FLX Titanium
MIGS-31.2	Sequencing coverage	309.5 × Illumina; 36.0 × pyrosequence
MIGS-30	Assemblers	Newbler version 2.3, Velvet 1.0.13, phrap version SPS - 4.24
MIGS-32	Gene calling method	Prodigal
	INSDC ID	PAHKR00000000
	GenBank Date of Release	January 24, 2012
	GOLD ID	Gc04190
	NCBI project ID	50579
	Database: IMG-GEBA	2506783053
MIGS-13	Source material identifier	DSM 15749
	Project relevance	Tree of Life, GEBA

### Growth conditions and DNA isolation

*G. limnaea* strain R-8282^T^, DSM 15749, was grown in DSMZ medium 514 (BACTO Marine Broth) [37] at 20°C. DNA was isolated from 0.5-1 g of cell paste using MasterPure Gram Positive DNA Purification kit (Epicentre MGP04100) following the standard protocol as recommended by the manufacturer with modification st/DL as described by Wu *et al*. 2009 [36] for optimized cell lysis. DNA is available through the DNA Bank Network [38].

### Genome sequencing and assembly

The genome was sequenced using a combination of Illumina and 454 sequencing platforms. All general aspects of library construction and sequencing can be found at the JGI website [39]. Pyrosequencing reads were assembled using the Newbler assembler (Roche). The initial Newbler assembly consisting of 93 contigs in one scaffold was converted into a phrap [40] assembly by making fake reads from the consensus, to collect the read pairs in the 454 paired end library. Illumina GAii sequencing data (1,096.5Mb) was assembled with Velvet [41] and the consensus sequences were shredded into 1.5 kb overlapped fake reads and assembled together with the 454 data. The 454 draft assembly was based on 178.7 Mb 454 draft data and all of the 454 paired end data. Newbler parameters are -consed -a 50 -l 350 -g -m -ml 20. The Phred/Phrap/Consed software package [40] was used for sequence assembly and quality assessment in the subsequent finishing process. After the shotgun stage, reads were assembled with parallel phrap (High Performance Software, LLC). Possible mis-assemblies were corrected with gapResolution [39], Dupfinisher [42], or sequencing cloned bridging PCR fragments with subcloning. Gaps between contigs were closed by editing in Consed, by PCR and by Bubble PCR primer walks (J.-F. Chang, unpublished). A total of 893 additional reactions and one shatter library were necessary to close gaps and to raise the quality of the final sequence. Illumina reads were also used to correct potential base errors and increase consensus quality using the software Polisher developed at JGI [43]. The error rate of the final genome sequence is less than 1 in 100,000. Together, the combination of the Illumina and 454 sequencing platforms provided 127.9 x coverage of the genome. The final assembly contained 597,282 pyrosequence and 33,599,185 Illumina reads.

### Genome annotation

Genes were identified using Prodigal [44] as part of the Oak Ridge National Laboratory genome annotation pipeline, followed by a round of manual curation using the JGI GenePRIMP pipeline [45]. The predicted CDSs were translated and used to search the National Center for Biotechnology Information (NCBI) non-redundant database, UniProt, TIGRFam, Pfam, PRIAM, KEGG, COG, and InterPro databases. These data sources were combined to assert a product description for each predicted protein. Additional gene prediction analysis and functional annotation was performed within the Integrated Microbial Genomes - Expert Review (IMG-ER) platform [46].

## Genome properties

**Figure 3:**
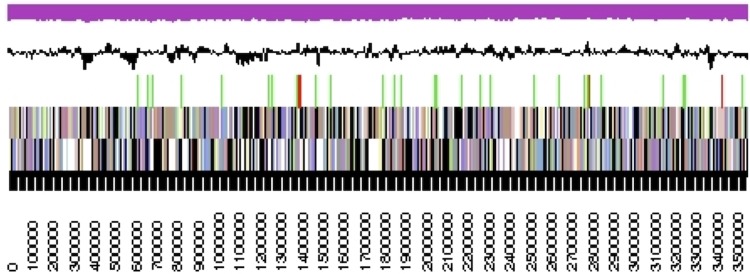
Graphical map of the largest scaffold. From bottom to top: Genes on forward strand (colored by COG categories), Genes on reverse strand (colored by COG categories), RNA genes (tRNAs green, rRNAs red, other RNAs black), GC content(black), GC skew (purple/olive).

The genome consists of two scaffolds with 3,558,876 bp and 407,981 bp length, respectively, with a G+C content of 37.6% (Table 3 and Figure 3). Of the 3,620 genes predicted, 3,569 were protein-coding genes, and 51 RNAs; 135 pseudogenes were also identified. The majority of the protein-coding genes (66.0%) were assigned a putative function while the remaining ones were annotated as hypothetical proteins. The distribution of genes into COGs functional categories is presented in Table 4.

**Table 3:** Genome Statistics

**Attribute**	**Value**	**% of Total**
Genome size (bp)	3,966,857	100.00%
DNA coding region (bp)	3,414,922	85.09%
DNA G+C content (bp)	1,490,901	37.61%
Number of scaffolds	2	
Total genes	3,620	100.00%
RNA genes	51	1.41%
rRNA operons	1*	
tRNA genes	44	1.22%
Protein-coding genes	3,569	98.59%
Pseudo genes	135	3.73%
Genes with function prediction (proteins)	2,388	65.97%
Genes in paralog clusters	1,727	47.71%
Genes assigned to COGs	2,489	68.76%
Genes assigned Pfam domains	2,606	71.99%
Genes with signal peptides	867	23.95%
Genes with transmembrane helices	826	22.82%
CRISPR repeats	0	

* one 23S rRNA gene, two 16S rRNA genes

**Table 4:** Number of genes associated with the general COG functional categories

**Code**	**value**	**%age**	**Description**
J	165	6.1	Translation, ribosomal structure and biogenesis
A	0	0.0	RNA processing and modification
K	148	5.5	Transcription
L	159	5.9	Replication, recombination and repair
B	1	0.0	Chromatin structure and dynamics
D	28	1.0	Cell cycle control, cell division, chromosome partitioning
Y	0	0.0	Nuclear structure
V	70	2.6	Defense mechanisms
T	125	4.7	Signal transduction mechanisms
M	277	10.3	Cell wall/membrane biogenesis
N	12	0.5	Cell motility
Z	0	0.0	Cytoskeleton
W	0	0.0	Extracellular structures
U	49	1.8	Intracellular trafficking and secretion, and vesicular transport
O	105	3.9	Posttranslational modification, protein turnover, chaperones
C	125	4.7	Energy production and conversion
G	146	5.4	Carbohydrate transport and metabolism
E	217	8.1	Amino acid transport and metabolism
F	61	2.3	Nucleotide transport and metabolism
H	131	4.9	Coenzyme transport and metabolism
I	94	3.5	Lipid transport and metabolism
P	131	4.9	Inorganic ion transport and metabolism
Q	59	2.2	Secondary metabolites biosynthesis, transport and catabolism
R	336	12.5	General function prediction only
S	250	9.3	Function unknown
-	1,131	31.2	Not in COGs

## Insights into the genome sequence

Genome analysis of *G. limnaea* R-8282^T^ revealed the presence of three rhodopsin genes related to proteorhodopsin (PR, GenBank Accession No. EHQ04368, Gilli_0216) and xanthorhodopsin (XR, EHQ02967, Gilli_2340) protein-encoding sequences, whereas a third rhodopsin protein sequence (EHQ02971, Gilli_2344) seems to be truncated. Another finding was a set of genes involved in β-carotene biosynthesis, together with a gene encoding a β-carotene 15,15'-monooxygenase (EHQ04367, Gilli_0215), an enzyme that oxidatively cleaves β-carotene into two molecules of retinal, which is necessary for rhodopsin function. PRs and XRs are photoactive transmembrane opsins that bind retinal and which belong to the microbial rhodopsin superfamily [47]. When exposed to light, a change in protein conformation causes a proton translocation with respect to its cofactor retinal from the inside to the outside of the cell [48]. This proton-pump activity generates a proton motive force across the cell membrane, which can be used in heterologously PR-expressing *E. coli* cells for ATP synthesis [49] as well as to power general cellular functions like transmembrane nutrient transport or flagella rotation [50]. In contrast to PRs, XRs are light-driven proton pumps containing a dual chromophore: one retinal molecule and one carotenoid antenna [51,52], that was first discovered in *Salinibacter ruber* M31^T^ [53,54]. Its carotenoid antenna salinixanthin transfers as much as 40-45% of the absorbed photons to retinal [55], resulting in a potentially much more efficient light capturing system compared to PRs from *Bacteria* [56,57] or bacteriorhodopsins from *Archaea* [58].

**Figure 4:**
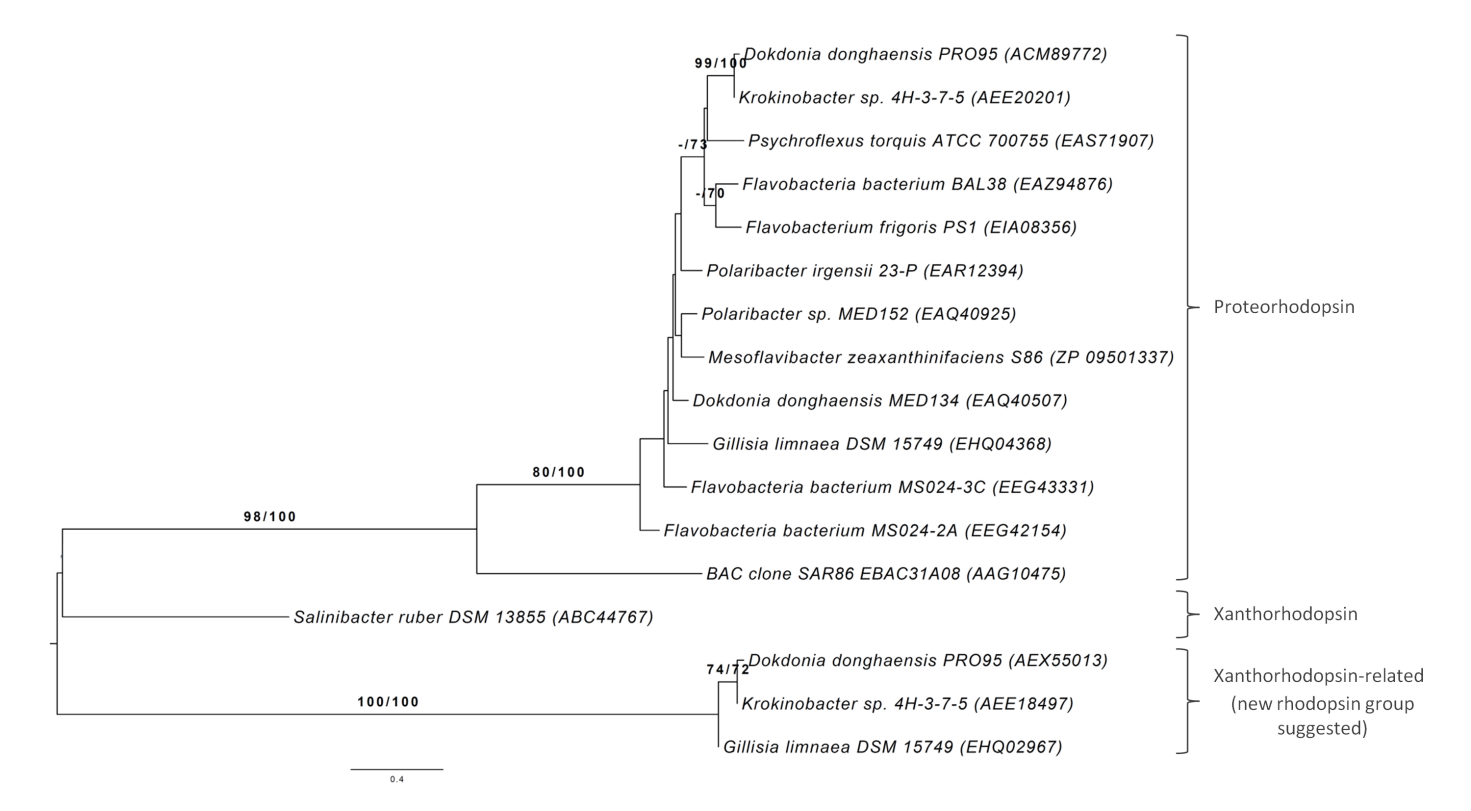
Rhodopsin tree for *Gillisia* and relatives. Amino acid sequences were processed in the same way as the 16S rRNA sequences used in Figure 1 except for the explicit determination of an optimal maximum-likelihood model, which turned out to be Lateral Gene Transfer [59]. GenBank Accession Numbers are shown in parentheses.

NCBI BLAST analysis [3] revealed that the protein encoded by Gilli_0216 shares distinct identities with many PR protein sequences, found in other species within the *Flavobacteriaceae* (Figure 4). It shows typical features necessary for proton pump activity: K224 (K231) for retinal-binding, and D88 (D97) as well as E99 (E108) (EBAC31A08 numbering shown in brackets), which act as a proton acceptor and proton donor in the retinylidene Schiff’s base transfer during the PR photocycle [60,61]. Furthermore, the putative PR (Gilli_0216 protein) has a M96 (L105) (EBAC31A08 numbering in parentheses), which mainly indicates that it is a green light-absorbing proteorhodopsin [48,62].

The gene encoding the putative XR (Gilli_2340) of strain R-8282^T^ shows identities to XR-related proteins, but provides evidence of a new cluster of rhodopsins found in very few flavobacterial isolates like *Dokdonia donghaensis* PRO95 (EHQ04368) [63] and *Krokinobacter* sp. 4H-3-7-5 (AEE18495) [64], which was reclassified into the genus *Dokdonia* [65,66] (Figure 4). This rhodopsin-encoding sequence also reveals typical features necessary for rhodopsin function: K316 (K231) for retinal binding and L181 (L105), which mainly indicates a green-light absorbing rhodopsin [48,62] (EBAC31A08 numbering shown in brackets). But amino acid residues functioning as proton acceptor and proton donor in proteorhodopsin differ from those commonly known. Instead of D97 and E108 (EBAC31A08 numbering), the related amino acids N173 and Q184 are found in the protein sequence encoded by Gilli_2340, which indicates a possible new kind of rhodopsins.

Interestingly, no rhodopsin-encoding sequence could be detected in the genome sequence of *Gillisia* sp. strain CBA3202 [67], which was isolated from the littoral zone on Jeju Island, Republic of Korea [67]. Digital DNA-DNA hybridization (DDH) [68] between strain R-8282^T^ and CBA3202 revealed an estimate between 9.7% and 13.9% (depending on the formula used), indicating that *Gillisia* sp. strain CBA3202 does not belong to the species *G. limnaea*.

Compared to free-living bacteria, representatives of the *Bacteroidetes* phylum were frequently found attached to aggregates [69] and during an algae-bloom collapse [70,71]. They were also known to move over surfaces by gliding motility [72,73]. In strain R-8282^T^ several genes were detected that are thought to be involved in gliding motility (*gld*A (Gilli_1140), *gld*B (Gilli_2923), *gld*C (Gilli_2942), *gld*D (Gilli_1840), *gld*E (Gilli_1841), *gld*F (Gilli_3447), *gld*G (Gilli_3446), *gld*H (Gilli_2158), *gld*I (Gilli_0258), *gld*J (Gilli_1638), *gld*K (Gilli_2747), *gld*L (Gilli_2748), *gld*M (Gilli_2749), *gld*N (Gilli_2750), *esp*A (Gilli_3049), *esp*B (Gilli_3050), *rem*B (Gilli_2697), *spr*A (Gilli_2693) and *spr*E (Gilli_2130)). This observation indicates the possible gliding motility of strain R-8282^T^, but has never been reported in literature.
